# From nuclear submarines to graduate medical education: applying David Marquet’s intent-based leadership model

**DOI:** 10.1186/s40779-017-0140-7

**Published:** 2017-10-11

**Authors:** Camilo Fernandez-Salvador, Rebecca Oney, Sungjin A. Song, Macario Camacho

**Affiliations:** 1 Department of Otolaryngology, Tripler Army Medical Center, Honolulu, 96859 Hawaii USA; 2 Otolaryngology-Head and Neck Surgery, Tripler Army Medical Center, 1 Jarrett White Road, Honolulu, 96859 Hawaii USA

**Keywords:** David Marquet, Leadership, Graduate medical education, Intent-based leadership

## Abstract

L. David Marquet, a decorated Navy Captain, transformed an underperforming submarine crew by empowering his subordinates to be leaders and reach their full potential. He called this intent-based leadership (IBL). What would happen if Marquet’s model were implemented in Graduate Medical Education (GME)?

In this letter to the editor, we summarize the potential of the IBL model in graduate medical education as opposed to the traditional leader-follower method. IBL harnesses human productivity toward the shared goals of GME, which are patient care and trainee learning. This shift in mindset could lead both teachers and trainees to focus more on the real reason that we undertake GME and change behaviors for the better. We suggest that IBL can and should be adopted in GME and propose that both patients and providers will benefit from this action.

## Dear editors:

L. David Marquet was a man on the rise. He was a top graduate of the Naval Academy class of 1981 and was poised to assume command of the nuclear-powered attack submarine, the USS Olympia. He had been preparing diligently for this role for more than a year when plans changed at the last minute. Instead, Marquet would unexpectedly become Captain of the USS Santa Fe, a different type of submarine, which had the worst performance record in the fleet.

A few weeks later, Captain Marquet would discover how rough the waters around him really were. He gave an order during a drill, “ahead two-thirds.” This order was passed down the line, but when it reached the helmsman who was supposed to execute the order, nothing happened. Although this was a valid order on other ships, this was not one that could be executed on the USS Santa Fe. When questioned, the officer on deck said that he had repeated the order, knowing that it was wrong. Marquet knew something had to change.

Marquet recognized the danger of the classic leader-follower model that was commonly practiced in the military and elsewhere. His crew would blindly follow his commands even if they knew that these orders were unsound. He boldly turned his back on the conventional leader-follower paradigm and employed a novel model in which each crewmember engaged and contributed to his full intellectual capacity. He empowered his subordinates to be leaders, allowing each person to feel valued and to reach his full potential. He called this IBL [1].

The results were astounding. “Santa Fe went from ‘worst to first,’ achieving the highest retention and operational standings in the Navy.” Even after Marquet left the Santa Fe, it continued to thrive, winning awards and promoting “more officers and enlisted men with increased responsibility than any other submarine”.

Can GME similarly benefit from IBL? We believe that the answer is yes. A search in the database, including PubMed and Google Scholar from February 1, 2015 through May 19, 2017 did not uncover values to measure the outcomes of IBL in GME. To our knowledge, this is the first letter to the editor exploring this idea.

The foundation of GME has historically been built on hierarchy, with numerous and often palpable delineations of power dividing the most experienced staff from the most junior medical students. Although this hierarchy provides structure, in some cases, it can stifle creativity and communication and can reduce intelligent, compassionate, once-inspired people to feel like slaves to the healthcare system.

In the traditional leader-follower paradigm, the leader is empowered to make decisions while the follower must simply take orders. The leader’s goals, however ill-defined or poorly understood by the follower, become the goals of the follower. For example, a medical student does not know why he is looking up the latest sodium level of a patient or getting a suture-removal kit for rounds, but does so anyway because he was told to. An intern follows an instruction to admit a patient and order a specific consult without understanding the overall plan of care. Although acceptable patient care can be accomplished using the leader-follower model, opportunities for deeper learning and a greater sense of purpose are lost.

The IBL model is not based on the flow of power from one individual to another as in the leader-follower model, but is instead based on a goal, or intent, shared between individuals. A military analogy is that the leader-follower model is similar to command and control, but the IBL model is similar to mission command. IBL allows an individual to feel positive about his/her role in the larger system. A resident trained in an IBL-driven environment would feel empowered to care for patients and learn his/her craft above all other purposes. With IBL, an individual is motivated by the intent itself rather than being motivated to meet a minimum standard as outlined by his/her superior. This subtle shift in mindset can dramatically alter how an individual conducts his/her job and understands his/her purpose in GME. Ultimately, it can enhance the quality of care that patients receive.

There are already examples of improvements in GME that are consistent with the IBL philosophy, although the term IBL has not previously been applied. Work hour restrictions that protect residents from situations in which labor is valued over education represent one example. The purpose of residency is the training of compassionate, competent physicians, and work hour restrictions represent a policy change that supports this intent. The implementation of the surgical time-out is another example. Every person in the operating room is given an opportunity to voice patient safety concerns, regardless of his/her status as a doctor, student, nurse, or technician. The intent of surgery is to safely complete a procedure for a patient, and the time-out serves as a reminder of this. Morning huddles in the clinical setting represent an opportunity for IBL. The leader of the morning huddle can empower each person to be individually accountable for his/her role in the care of patients, such as the effective utilization of resources and the maintenance of a timely schedule. When everyone has a shared goal and feels empowered to pursue it, the system can work smoothly and both patients and providers can benefit.

The full adoption of IBL in GME would first require a clear definition of the intent of GME, which we believe includes optimal patient care and optimal trainee learning. Leaders at every level of GME would then need to embrace this shared intent, adopting a goal-oriented attitude and training those below them with encouragement. Senior residents and staff would serve as mentors to those below them rather than maintaining a domineering relationship. Learners could abandon the excuse of “I’m only a medical student, only an intern” and instead orient their efforts toward the accomplishment of excellent patient care and maximal learning.

If IBL were applied in a way that compromised oversight or patient safety standards, there could be negative implications for patients and trainees. Therefore, it would be important for trainees to know the appropriate bounds of their actions and feel comfortable asking for help when needed.

IBL is not a theoretical idea - it involves the ubiquitous transformation of high-functioning systems. We suggest that IBL should be adopted in GME in most situations and propose that both patients and physicians will benefit.

## Abbreviations

GME: Graduate medical education
IBL: Intent-based leadership
